# Retrospective analysis of the transmission of difficult-to-treat resistant *Pseudomonas aeruginosa* – genotype beats phenotype

**DOI:** 10.1186/s13756-026-01851-3

**Published:** 2026-10-30

**Authors:** Jasmin Kaur Jasuja, Philipp Maximilian Maurer, Laura Carlsen, Eva-Maria Theresia Klupp, Cristina Elena Belmar Campos, Holger Rohde, Martin Aepfelbacher, Johannes Karl-Mark Knobloch

**Affiliations:** 1 Department for Infection Prevention and Control, Institute for Microbiology, Virology and Hygiene, University Medical Center Hamburg-Eppendorf, Martinistraße 52, 20246 Hamburg, Germany; 2 Institute for Medical Microbiology, Virology and Hygiene, University Medical Center Hamburg-Eppendorf, Martinistraße 52, 20246 Hamburg, Germany

**Keywords:** Difficult-to-treat *Pseudomonas aeruginosa*, Molecular surveillance, Outbreak management, Infection prevention and control, Whole genome sequencing

## Abstract

**Background:**

Difficult-to-treat resistant *Pseudomonas aeruginosa* (DTRPA) represents a challenging resistance phenotype with carbapenem-resistant *P. aeruginosa* playing a major role. However, carbapenemase-negative DTRPA (CN-DTRPA) are more susceptible to reserve antibiotics and rarely reported in outbreaks, differing from frequently outbreak-associated carbapenemase-producing DTRPA (CP-DTRPA) with limited therapeutic options. Despite, international guidelines recommend equal infection prevention and control (IPC) measures. Here, we analysed the transmission of CP-DTRPA and CN-DTRPA using epidemiology and whole-genome sequencing (WGS).

**Methods:**

Clinical DTRPA isolates collected between 2019 and 2024 were subjected to WGS. Genetic clusters were defined using cgMLST thresholds of ≤ 11 and ≤ 3 allelic differences. Epidemiological relatedness was estimated on hospital stay, interpatient contact and environmental exposures. For possibly epidemiologically linked cases, precolonisation with carbapenem-susceptible *P. aeruginosa* (CSPA) and antibiotic exposure during hospitalisation were assessed.

**Results:**

Among 190 DTRPA isolates, CN-DTRPA (79.5%) was predominant compared to 20.5% CP-DTRPA, mainly carrying *bla*_VIM−2_. Twelve genetic clusters involving 39 patients were identified. Seven clusters (CP-DTRPA *n* = 4; CN-DTRPA *n* = 2, CP-DTRPA/CN-DTRPA-mixed *n* = 1) showed possible epidemiological relatedness. CP-DTRPA isolates were frequently associated with clusters (OR 17.47, 95% CI 7.43–41.08) and environmental transmission. Diverse CN-DTRPA were rather linked to interpatient CSPA-transmission following CN-DTRPA emergence after prolonged antibiotic exposure.

**Conclusion:**

CN-DTRPA predominated and displayed high diversity, consistent with possible antibiotic-driven resistance development in precolonised patients. In contrast, less frequent and less diverse CP-DTRPA were associated to outbreaks. These findings highlight the need to evaluate differentiated IPC strategies, including targeted screening and environmental interventions for CP-DTRPA, while further investigating the potential role of antimicrobial stewardship in preventing the emergence of CN-DTRPA. Multicentre studies are required for evidence-based IPC strategies.

**Supplementary Information:**

The online version contains supplementary material available at https://doi.org/10.1186/s13756-026-01851-3.

## Introduction

Carbapenem-resistant *Pseudomonas aeruginosa* represents a major part of difficult-to-treat resistant *P. aeruginosa* (DTRPA), a phenotype defined by non-susceptibility to all standard antipseudomonal β-lactams, fluoroquinolones, and carbapenems [1]. Under antibiotic selective pressure, *P. aeruginosa* can rapidly develop resistance arising from both carbapenemase production and intrinsic mechanisms, including AmpC β-lactamase overexpression, efflux pump activation and porin loss, ultimately resulting in DTRPA [2]. However, there are important molecular and clinical distinctions between carbapenemase-producing (CP-DTRPA) and carbapenemase-negative (CN-DTRPA) DTRPA. CP-DTRPA are frequently reported in internationally disseminated high-risk clones that have been implicated in numerous hospital outbreaks worldwide [3]. These clones are commonly detected during environmental screening performed as part of outbreak investigations, underscoring their ability to persist and spread in sinks [4, 5]. CP-DTRPA harbour carbapenemase genes located on mobile genetic elements, which facilitate horizontal gene transfer and clonal expansion [2]. In line with this, whole-genome sequencing (WGS) studies have demonstrated a strong association between carbapenemase production and clonal clustering linked to large and prolonged outbreaks [4, 5].

In contrast, CN-DTRPA isolates are generally more susceptible to reserve antibiotics and are reported less frequently in outbreak settings [6]. When outbreaks occur, they tend to be smaller, though allelic difference is considered small [5]. However, the source and transmission route of CN-DTRPA remain poorly understood [5]. Prolonged transmission chains, when observed, have occasionally been traced back to contaminated medical products [7]. It is unclear whether the absence of carbapenemase production translates into a fundamentally different transmission behaviour comparable to carbapenem-susceptible *P. aeruginosa* (CSPA) or still resembles to CP-DTRPA.

Despite, most international infection prevention and control (IPC) guidelines apply identical measures to both CP-DTRPA and CN-DTRPA [8–11]. Such measures often include resource-intensive interventions, such as patient isolation and enhanced environmental cleaning. Only a limited number of international guidelines explicitly differentiate between CP-DTRPA and CN-DTRPA, underlining the molecular difference and restrict enhanced IPC measures to CP-DTRPA alone (Supplementary Table 1) [12–14]. Despite the globally reported widespread of CP-DTRPA high-risk clones in hospital environment, the heterogeneity in international guidelines highlights the inconsistency in current IPC recommendations. This raises the important question of whether IPC strategies should differentiate between CP-DTRPA and CN-DTRPA.

WGS provides detailed insights into transmission pathways that cannot be captured by conventional epidemiological approaches alone. Hence, this study analyses the transmission of CP-DTRPA and CN-DTRPA by combining epidemiological data with WGS over a six-year surveillance period to evaluate their respective transmission potential and to inform evidence-based IPC strategies.

## Materials and methods

### Study design and setting

This study was conducted between January 2019 and December 2024 at a tertiary care hospital in Germany. During the aforementioned period all DTRPA isolates defined according to German healthcare authorities with (i) non-susceptibility to all standard antipseudomonal β-lactams, fluoroquinolones and carbapenems, (ii) phenotypic resistance to carbapenems and resistance to two of three aforementioned antibiotic groups or (iii) molecular detection of carbapenemase were obtained from routine clinical and screening samples from in- and outpatients and considered for WGS [11]. Paediatric samples were excluded as well as isolates phenotypically and genetically susceptible to both imipenem and meropenem. For carbapenem susceptibility clinical breakpoints were taken from the respective EUCAST recommendations (2019–2024). One sample per patient was included. In case of multiple sequenced isolates of a single patient, preferably first available DTRPA isolate detected during the aforementioned study period was subjected to further analysis.

### Whole-genome sequencing

Genomic DNA was extracted using the MagNA Pure 96 Systems (Roche Diagnostics, Mannheim, Germany). WGS using the NextSeq500 platform (Illumina, San Diego, USA) was performed as previously described [15, 16]. Sequencing quality was evaluated by coverage depth and assembly completeness. De novo assembly was carried out using the integrated Velvet assembler (Version 1.1.04) within SeqSphere+ software (Bruker MBioSEQ Ridom Typer, Bremen, Germany, Version 11.0.0). MLST and sequence type (ST) determination was performed using the *P. aeruginosa* PubMLST database [17]. cgMLST was performed using the published *P. aeruginosa* cgMLST scheme [18]. Isolates with insufficient coverage (< 90%) in core genome multilocus sequence typing (cgMLST) were excluded from further analysis. Clusters were defined as groups of isolates differing by ≤ 11 alleles, consistent with previously published thresholds for *P. aeruginosa* [15, 19]. To identify highly related cluster continuing analysis included ≤ 3 allelic differences only [15]. Resistance gene detection was performed using AMRFinderPlus (National Center for Biotechnology Information, Version 4.0.15) [20].

### Epidemiological investigation

Clusters identified by cgMLST were further analysed using detailed epidemiological data. Epidemiological relatedness was defined as any possible epidemiological link between patients before or during the period when DTRPA was detected. This included potential exposure to shared environmental reservoirs (e.g., sinks, surfaces), irrespective of overlapping hospital stays, and/or possible direct or indirect contact between patients with overlapping hospitalisation. Further, epidemiological relatedness was distinguished in two subcategories, namely (i) potential interpatient transmission, defined as overlapping hospitalisation on the same ward for at least 24 h, with either co-location in the same patient room or potential indirect contact, such as shared healthcare personnel or medical equipment (e.g. ultrasound devices) during the time DTRPA was detected, and in (ii) probable environment-to-patient transmission, defined as DTRPA acquisition linked to exposure to common environmental sources in the same building without overlapping hospital stay or (in)direct patient contact.

DTRPA recovered from environmental sampling were either in the context of targeted outbreak investigations or as incidental findings during routine, non–DTRPA-targeted environmental sampling. DTRPA isolates from potential environmental sources were identified months to years before and/or after the detection of corresponding outbreak DTRPA isolates. Only environmental DTRPA isolates carrying a carbapenemase gene were subjected to WGS.

Non-clinical, epidemiological relevant data and previous microbiological results of patients with isolates involved in clusters were reviewed to identify prior colonisation or infection with CSPA. CSPA was defined as *P. aeruginosa* phenotypically susceptible to at least imipenem and meropenem. Isolates further susceptible to piperacillin, ceftazidime and ciprofloxacin, respectively, were also considered as CSPA. These CSPA isolates were not available for sequencing. Further, medical records were investigated on antipseudomonal antibiotic therapy (imipenem, meropenem, piperacillin/tazobactam, ceftazidime, cefepime, ciprofloxacin), cumulative days of *Pseudomonas*-effective and non-effective (all others except aforementioned antibiotics) antibiotic therapy and number of applied antibiotic agents given during the hospital stay in which DTRPA identification occurred until the day of DTRPA detection limited to cluster-associated CN- and CP-DTRPA.

### Ethical consideration

In view of the fact that the patient data that is the subject of the study can no longer be attributed to a human being, the study project does not constitute a ‘research project involving human beings’ as defined in Section 9 (2) of the Hamburg Chamber Act for the Medical Professions and also does not fall within the scope of the research projects requiring consultation pursuant to Section 15 (1) of the Professional Code of Conduct for Hamburg Physicians. Thus, this study project does not require consultation with the Ethics Committee of the Hamburg Medical Association (file number: 2025-300624-WF). As an IPC routine task, clusters were investigated on transmission paths. For publication, sensitive data were anonymised.

## Results

**Fig. 1:**
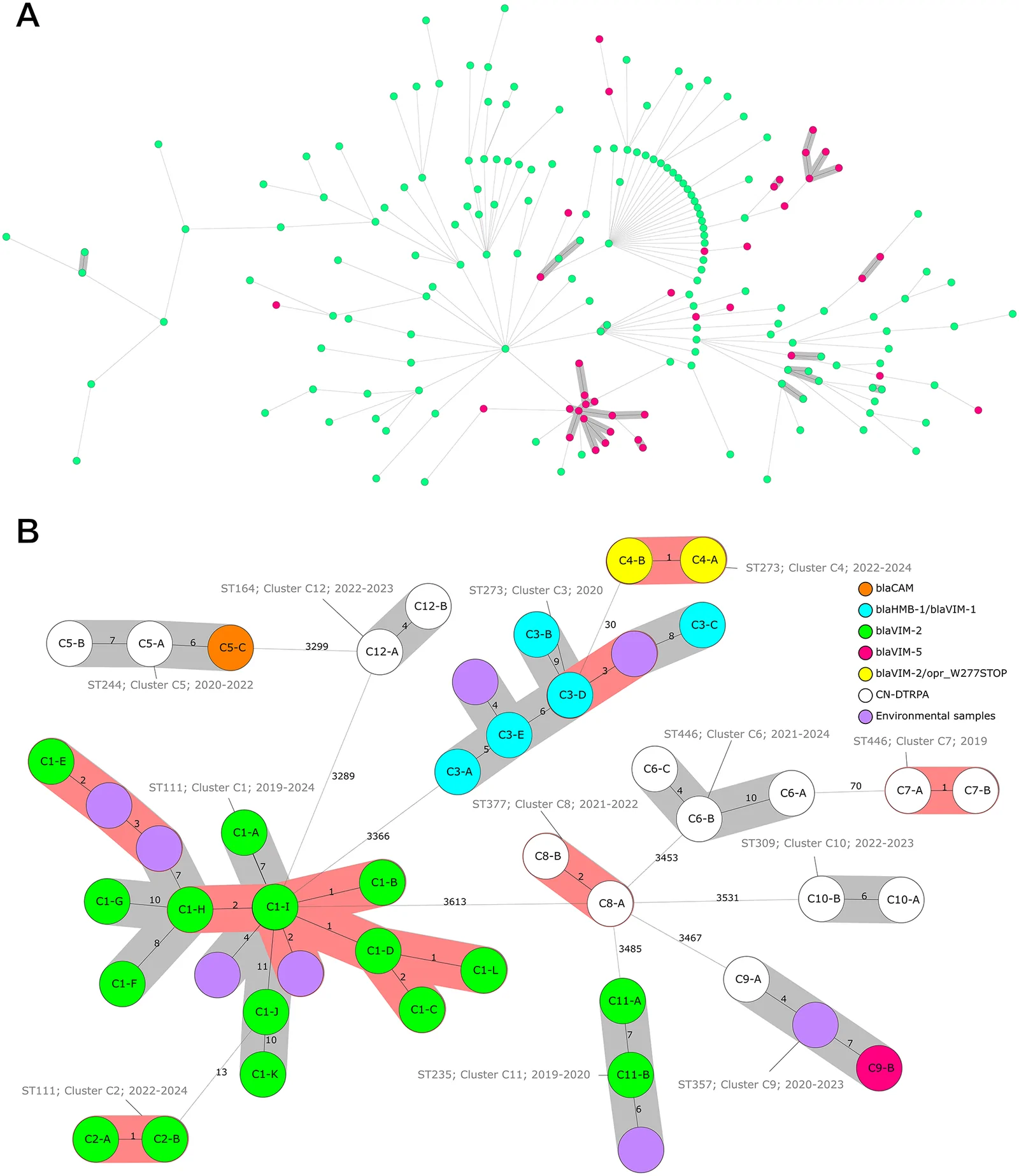
(**A**) Overview of 190 difficult-to-treat resistant *Pseudomonas aeruginosa* (DTRPA) analysed on transmission dynamics of carbapenemase-producing (CP-) (pink) and carbapenemase-negative (CN-)DTRPA (green) in a tertiary care hospital in Germany. (**B**) Overview of all twelve clusters comprising 39 DTRPA isolates in a study of a six-year surveillance on transmission dynamics of CP- and CN-DTRPA. Thereof, twelve clusters (grey surrounding) with ≤ 11 allelic differences were found. Applying a cut-off of ≤ 3 allelic differences, five highly related clusters were identified (red surrounding). The purple circles represent environmental samples. All environmental samples were carbapenemase-producing. The carbapenemase matched to the CP-DTRPA cluster. In case of a mixed C-/CP-DTRPA cluster, the carbapenemase matched to the CP-DTRPA isolate

During the six-year-surveillance period, a total of clinical 193 non-duplicate DTRPA were sequenced. Thereof, three isolates were excluded because of insufficient coverage. A total of 190 (98.4%) isolates were available for final analysis. Most isolates were CN-DTRPA (*n* = 151; 79.5%), while 39 (20.5%) were CP-DTRPA. The most frequent carbapenemase genes were *bla*_VIM−2_ (*n* = 23), followed by *bla*_HMB−1_ (*n* = 7), *bla*_VIM−1_ (*n* = 6), though *bla*_HMB−1_ was frequently detected in dual combination with *bla*_VIM−1_ (*n* = 5), and *bla*_NDM−1_ (*n* = 3) (Supplementary Table 2). A total of 98 STs were identified, including eleven new found STs (ST6711, ST6719, ST6726, ST6747, ST6765, ST6766, ST6767, ST6768, ST6769, ST6778 and ST6684). Meanwhile for three isolates no ST could be assigned, each due to one missing allele. The non-identified STs did not belong to clusters and were not single locus variant to other STs. In CP-DTRPA a total of twelve STs were identified with ST111 (*n* = 15) and ST273 (*n* = 8) being predominant and five ST singletons. In CN-DTRPA 94 STs were identified with ST244 (*n* = 7), ST446 (*n* = 6) and ST395 (*n* = 6) being observed most frequently and 71.3% singletons. CN-DTRPA exhibited substantially higher genetic diversity than CP-DTRPA, as quantified by Simpson’s diversity index (0.99 vs. 0.79), reflecting a predominance of singletons among CN-DTRPA (Fig. 1A).

Applying a clustering threshold of ≤ 11 allelic differences, twelve clusters comprising CP-DTRPA and CN-DTRPA among 39 DTRPA isolates were identified (Fig. 1B): five CP-DTRPA clusters (C1, C2, C3, C4, C11), five CN-DTRPA clusters (C6, C7, C8, C10, C12), and two mixed clusters containing both CP- and CN-DTRPA (C5, C9) (Table 1). CP-DTRPA isolates were significantly more likely to be associated with genetic clusters than CN-DTRPA isolates (CP-DTRPA 25/39 vs. CN-DTRPA 14/151; Fisher’s exact test, *p* < 0.001; OR 17.47, 95% CI 7.43–41.08). This association remained robust after the exclusion of the large ST111 cluster C1 (*n* = 12) with 13/27 CP-DTRPA (without ST111 C1-cluster) compared with 14/151 CN-DTRPA associated with genetic clusters (OR 9.09, 95% CI 3.57–23.12; Fisher’s exact test, *p* < 0.0001). For seven clusters (C1, C2, C3, C4, C7, C8, C9) possible epidemiological linkage could be defined (described in detail below; remaining clusters see Table 1).

**Table 1:** Overview of potential transmission risk of difficult-to-treat resistant *P. aeruginosa* (DTRPA) in six years surveillance study on carbapenemase-positive (CP-DTRPA) and carbapenemase-negative *P. aeruginosa* (CN-DTRPA) in a tertiary care hospital in Germany. Epidemiological relatedness: any plausible link during DTRPA detection, via (i) interpatient-transmission (≥ 24 h overlap on same ward with direct/indirect contact) or (ii) environment-to-patient transmission (shared sources, e.g., sinks, healthcare personnel) in the same building without hospital overlap

Cluster-ID	ST	Cluster size (*n*)	DTRPA type	Prior colonisation with CSPA	Year(s) of DTRPA detection	Epidemiological relatedness	Interpatient transmission	Environment-to-patient transmission
C1	ST111	12	CP-DTRPA (*bla*_VIM-2_)	2/12	2019, 2020, 2022, 2023, 2024	Yes	No	Possible
C2	ST111	2	CP-DTRPA (*bla*_VIM-2_)	1/2	2022, 2024	Yes	No	Possible
C3	ST273	5	CP-DTRPA (*bla*_HMB-1_/ *bla*_VIM-1_)	1/5	2020	Yes	No	Possible
C4	ST273	2	CP-DTRPA (*bla*_VIM-2_)	0/2	2022, 2024	Yes	No	Possible
C5	ST244	3	CP-DTRPA/CN-DTRPA (*bla*_CAM_/negative)	2/3	2020, 2022	No	No	No
C6	ST446	3	CN-DTRPA	1/3	2021, 2022, 2024	No	No	No
C7	ST446	2	CN-DTRPA	1/2	2019	Yes	Possible	Possible
C8	ST377	2	CN-DTRPA	2/2	2021, 2022	Yes	Possible	Possible
C9	ST357	2	CP-DTRPA/CN-DTRPA (*bla*_VIM-5_/negative)	0/2	2020, 2023	Yes	No	Possible
C10	ST309	2	CN-DTRPA	2/2	2022, 2023	No	No	Possible
C11	ST235	2	CP-DTRPA (*bla*_VIM-2_)	0/2	2019, 2020	No	No	Possible
C12	ST164	2	CN-DTRPA	2/2	2022, 2023	No	No	Possible

Cluster C1 represented an intensive care unit (ICU) outbreak caused by high-risk clone *P. aeruginosa* ST111 harbouring *bla*_VIM−2_ [15]. Twelve patients were affected, but only six isolates differing by ≤ 3 alleles, were epidemiologically linked. The remaining isolates of the cluster (4–11 allelic differences) originated from patients without exposure to the affected ICU, though three were associated to the oncological department, and hence, were considered as not directly related to the outbreak. Within the confirmed outbreak environmental sampling identified *P. aeruginosa bla*_VIM−2_-positive isolates in two sinks in two ICU rooms, matching isolates from the six outbreak-associated patients and suggesting a possible sink-to-patient transmission [15].

Cluster C2, also belonging to *bla*_VIM−2_-positive ST111, involved two patients with a single allelic difference who had been admitted to the same ward two years apart. Although a 13 allele difference was defined with one isolate of C1, neither had an identifiable epidemiological link to C1. No environmental isolate could be linked to cluster C2.

In cluster C3 (ST273 carrying CP-DTRPA *bla*_HMB−1_/*bla*_VIM−1_), comprising five isolates from patients without overlapping hospitalisation, the allelic differences ranged from five to nine. However, all CP-DTRPA C3 cases occurred within two months across two wards (two in oncological ICU, two in oncological ward). Epidemiological investigation identified the first positive patient who was admitted to both wards. Environmental sampling during non-outbreak-associated time and prior and after potential transmission revealed two matching isolates belonging to cluster C3, one highly related (≤ 3 distinct alleles) to the first positive patient.

A second ST273 cluster (C4) with two highly related (one allelic difference) *bla*_VIM−2_ positive isolates represented two patients identified two years apart in the same ICU. No environmental isolate displayed clonal relation to these isolates.

Cluster C7 of two CN-DTRPA ST446 isolates had a single allelic difference indicating high relatedness (Fig. 1B). Detailed epidemiological investigation displayed, that both patients shared an ICU room for seven days. During this period, none of the patients displayed DTRPA. However, one patient carried a CSPA, while the second patient had no prior CSPA-colonisation before room sharing according to the microbiological database. The second patient got positive with a CN-DTRPA as detected after transfer to another ward. Both patients developed CN-DTRPA after treatment with antipseudomonal-active agents while admitted on different wards, almost two months after the last contact.

CN-DTRPA cluster C8 (ST377) comprised two highly related ICU patients (two allelic differences). The patients had overlapping ICU stays without room sharing. The first patient was placed under isolation precautions upon admission due to carbapenem-resistant *Acinetobacter baumannii* complex. This patient had a documented CSPA colonisation while staying on this ICU and displayed first CN-DTRPA detection one month after hospitalisation. The second patient was not isolated prior to CN-DTRPA detection and tested positive for CSPA colonisation three weeks after hospitalisation to this ICU. CN-DTRPA in the second patient occurred nearly three months after the first patient had died. In both patients, CSPA colonisation occurred during the overlapping stay at the ICU. Notably, CSPA colonisation in the second patient was detected nine days after the first patient’s initial CSPA detection.

Cluster C9 (ST357) involved two ICU patients identified three years apart, one with CP-DTRPA (*bla*_VIM−5_) and one with CN-DTRPA, differing by eight alleles. Carbapenemase-PCR was initially performed on the CN-DTRPA isolate and detected a *bla*_VIM_ carbapnemase gene, which was not detected by WGS. Non-targeted environmental sampling displayed a match between the environmental isolate carrying *bla*_VIM−5_ and both clinical isolates.

The remaining clusters (C5, C6, C10, C11, C12) with a maximum of three isolates included lacked clear epidemiological link, as none of the affected patients had spatiotemporal overlap with another. Notably, isolates in these clusters lacked high relatedness, as they had 4–11 allelic differences. Isolates in cluster C5 (ST244; seven allelic differences) were detected in different years and hospital buildings. Notably, two patients had prior CSPA colonisation, whereas this was lacking in the third patient harbouring a *bla*_CAM_ homologue gene. The CN-DTRPA cluster C6 (ST446; ≤10 allelic differences) comprised three isolates, which were found across three different buildings within three years. Only one patient had a CSPA precolonisation. Two patients with isolates in cluster C10 (ST309; six allelic differences) had a hospital stay in the same building but eleven months apart. Both patients had prior colonisation with CSPA and were admitted to the same department. Cluster C11 (ST235; seven allelic differences) carrying *bla*_VIM−2_ was detected in oncological patients within five months. Neither patient had prior colonisation with CSPA, and one patient was only seen as an outpatient. Non-targeted environmental sampling identified a matching isolate. The CN-DTRPA cluster C12 (ST164; four allelic differences) involved two patients admitted to different ICUs in the same building three months apart.

Most of the isolate’s patients belonging to clusters with epidemiological link received antipseudomonal antibiotics with ≥ 4 antibiotic agents (Table 2). CP-DTRPA clusters (*n* = 25) had an average of three different antibiotic agents and 36.2 days of antibiotic therapy. 15 of 25 CP-DTRPA-carrying patients (60.0%) were given meropenem during their hospital stay, while six patients (24.0%) were not treated with antibiotics at all. CN-DTRPA (*n* = 14) had an average of four different antibiotic agents and 53.0 days of antibiotic therapy. Eleven of 14 (78.6%) CN-DTRPA patients received meropenem, while a single patient (7.1%) did not receive any antibiotic therapy. Meropenem was more frequently administered to patients with CN-DTRPA than to those with CP-DTRPA (78.6% vs. 60.0%), although this difference was not statistically significant (Fisher’s exact test, *p* = 0.30).

**Table 2:** Antibiotic exposure (intravenous and oral) of patients within difficult-to-treat resistant *Pseudomonas aeruginosa* (DTRPA) clusters during the hospital stay in which DTRPA acquisition occurred. For each patient (cluster ID and patient ID), the cumulative number of treatment days with *Pseudomonas aeruginosa*-active antibiotics (imipenem, meropenem, piperacillin/tazobactam, ceftazidime, ciprofloxacin) is shown. Non-*P. aeruginosa*-active antibiotics (all others except aforementioned antibiotics) were not listed individually; cumulative treatment days with all antibiotics are provided in an additional column. Prophylactic antibiotic use, antifungal agents, and antivirals were not included. Data reflect only the period before DTRPA acquisition was detected

Cluster-ID	Patient-ID	DTRPA-Type	Imipenem (d)		Meropenem (d)	Piperacillin/ Tazobactam (d)	Ceftazidime (d)		Cefepime (d)	Ciprofloxacin (d)	Cumulative days of therapy of antibiotic therapy incl. non-*P*. aeruginosa-active therapy (d)	Number of different antibiotic agents received (*n*)
C1 (ST111)	C1-A	CP-DTRPA		-	Yes (9)	Yes (1)		-	-	-	10	2
	C1-B	CP-DTRPA		-	-	Yes (10)		-	-	-	10	1
	C1-C	CP-DTRPA		-	Yes (11)	Yes (6)		-	-	-	17	2
	C1-D	CP-DTRPA		-	Yes (15)	Yes (7)		Yes (2)	-	-	50	7
	C1-E	CP-DTRPA		-	-	Yes (15)		-	Yes (8)	Yes (11)	34	2
	C1-F	CP-DTRPA		-	-	-		-	-	-	-	-
	C1-G	CP-DTRPA		-	-	-		-	-	-	-	-
	C1-H	CP-DTRPA			-	-		-	-	-	-	-
	C1-I	CP-DTRPA		-	Yes (52)	-		Yes (16)	Yes (8)	-	105	8
	C1-J	CP-DTRPA		-	-	-		-	-	-	-	-
	C1-K	CP-DTRPA		-	Yes (30)	-		Yes (1)	-	-	67	5
	C1-L	CP-DTRPA		-	-	-		-	-	-	12	1
C2 (ST111)	C2-A	CP-DTRPA		-	Yes (22)	Yes (4)		Yes (1)	-	-	49	6
	C2-B	CP-DTRPA		-	-	Yes (2)		Yes (2)	-	-	11	4
C3 (ST273)	C3-A	CP-DTRPA		-	Yes (49)	-		Yes (27)	-	Yes (5)	163	7
	C3-B	CP-DTRPA		-	Yes (6)	Yes (4)		-	-	-	11	3
	C3-C	CP-DTRPA		-	-	-		-	-	-	-	-
	C3-D	CP-DTRPA		-	Yes (16)	-		Yes (6)	-	Yes (7)	50	5
	C3-E	CP-DTRPA		-	Yes (42)	-		-	-	Yes (4)	100	5
C4 (ST273)	C4-A	CP-DTRPA		-	Yes (31)	Yes (4)		-	-	-	41	3
	C4-B	CP-DTRPA		-	Yes (2)	-		-	-	-	2	1
C5 (ST244)	C5-A	CN-DTRPA		-	-	-		-	-	7	12	2
	C5-B	CN-DTRPA		-	Yes (18)	Yes (14)		-	-	-	44	4
	C5-C	CP-DTRPA		-	Yes (11)	Yes (8)		-	-	-	37	6
C6 (ST446)	C6-A	CN-DTRPA		-	Yes (1)	-		-	-	-	1	1
	C6-B	CN-DTRPA		-	-	-		-	-	-	-	-
	C6-C	CN-DTRPA		-	Yes (6)	-		-	-	-	8	2
C7 (ST446)	C7-A	CN-DTRPA		-	Yes (17)	Yes (41)		Yes (2)	-	Yes (1)	108	8
	C7-B	CN-DTRPA		-	Yes (20)	Yes (6)		Yes (14)	-	-	83	9
C8 (ST377)	C8-A	CN-DTRPA		-	Yes (22)	-		Yes (1)	Yes (2)	Yes (8)	88	8
	C8-B	CN-DTRPA		-	Yes (57)	Yes (7)		Yes (11)	Yes (14)	Yes (34)	164	6
C9 (ST357)	C9-A	CN-DTRPA		-	Yes (30)	-		Yes (15)	-	-	75	4
	C9-B	CP-DTRPA		-	Yes (54)	-		-	-	Yes (2)	102	5
C10 (ST309)	C10-A	CN-DTRPA		-	Yes (7)	Yes (5)		Yes (11)	-	-	32	4
	C10-B	CN-DTRPA		-	Yes (4)	-		-	-	-	4	1
C11 (ST235)	C11-A	CP-DTRPA		-	Yes (9)	-		-	-	-	34	3
	C11-B	CP-DTRPA		-	-	-		-	-	-	-	-
C12 (ST164)	C12-A	CN-DTRPA		-	Yes (3)	-		-	-	-	5	2
	C12-B	CN-DTRPA		-	-	Yes (13)		Yes (4)	Yes (21)	Yes (8)	118	8

## Discussion

Most international IPC guidelines apply equal IPC strategies to DTRPA despite possible different underlying resistance mechanism. In consideration of the different resistance patterns, we characterised the molecular epidemiology and potential transmission of CN- and CP-DTRPA over a six-year period in a tertiary care hospital to critically examine current recommendation in international IPC guidelines. CP-DTRPA, although overall less frequent, were frequently associated with transmission clusters, underscoring their higher transmission potential. In parallel, CP-DTRPA demonstrated lower genetic diversity, reflecting the dominance of high-risk clones ST111 and ST273. In four (C1, C2, C3, C4) of five CP-DTRPA clusters, environment-to-patient transmission seemed possible and, in some cases, possible environmental sources could be identified with clonally related isolates from targeted or non-targeted environmental sampling [15]. In contrast, CN-DTRPA displayed a higher diversity with 91 STs in a total of 151 isolates and were largely composed of ST singletons (71.3%). A possible interpatient transmission event was only plausible in two (C7, C8) of five CN-DTRPA clusters. Notably, in both clusters, patients were precolonised with CSPA, suggesting transmission in the phenotypic state of CSPA during overlapping hospital stays with subsequent emergence of CN-DTRPA under prolonged antibiotic exposure. The high genetic diversity and frequent occurrence as singletons may suggest that CN-DTRPA predominantly emerge through antibiotic-driven resistance development rather than clonal transmission, whereas CP-DTRPA could be primarily linked to patient-to-patient transmission and via persistent environmental reservoirs. However, this conclusion is limited by the lack of WGS data from environmental isolates associated with CN-DTRPA and therefore remains a hypothesis until further data are available.

Sinks have repeatedly been identified as a major global source of CP-DTRPA acquisition, particularly in ICUs, with transmission sometimes spanning multiple wards or occurring after long intervals, underscoring the remarkable persistence of environmental reservoirs as proven in modelling studies [15, 21–23]. High-risk clones such as ST111 and ST273 are globally well known for their outbreak potential involving multiple patients as demonstrated here [22, 24, 25]. This was underlined in the current study by further identified high-risk clones ST244 and ST357. However, both clusters C5 (ST244) and C9 (ST357) contained both CP-DTRPA and CN-DTRPA, though for C9 gene loss during subculturing prior WGS may explain the observed match with carbapenemase positive environmental isolates [26]. The predominance of *bla*_VIM−2_ among CP-DTRPA isolates in our cohort is in line with previous reports highlighting this carbapenemase gene as a key factor associated with outbreak persistence and limiting therapeutic options [27].

Our data align with previous studies demonstrating antibiotic pressure on precolonisation as a key driver of resistance development in CN-DTRPA rather than interpatient or environment-to-patient transmission [28]. In fact, up to 74% drug-resistant *P. aeruginosa* derive from a previous susceptible *P. aeruginosa* colonisation [29]. While prior hospitalisation and exposure to quinolones and carbapenems have been described as risk factors for DTRPA, differentiation between CP- and CN-phenotypes was often lacking [30]. In our cohort, meropenem was the most frequently administered antibiotic and was more commonly used in patients with CN-DTRPA. This pattern is biologically plausible, as resistance mechanisms in CN-DTRPA are typically mutational genes and regulations, respectively, and therefore more likely to emerge under antibiotic selection pressure rather than through horizontal gene transfer [2]. However, antibiotic, particularly meropenem, exposure rates were not significantly different and only covered the 39 cluster patients, while antibiotic exposure of residual 137 singletons dominated by CN-DTRPA were not investigated. Notably, interpatient transmission was considered only at the stage of CSPA colonization, whereas transmission of CN-DTRPA itself was not evident. The analysis of epidemiology seems rather important in CN-DTRPA clusters, as a study on imipenem-resistant *P. aeruginosa*, 31% of isolates appeared genetically related, but only 11% remained consistent with plausible transmission after combining molecular analysis with epidemiological data [31]. Hence, prevention efforts may not exclusively target resistant isolates, but rather the overall acquisition of *P. aeruginosa* by strict adherence to standard precautions, including the WHO ‘my five moments of hand hygiene’ and an established water safety plan as well as antibiotic stewardship (ABS) measures to reduce resistance development [5]. In the event of a CN-DTRPA outbreak, an environmental reservoir should be regarded as a plausible and relevant source, as demonstrated by a nationwide CN-*P. aeruginosa* outbreak in Norway due to a contaminated washcloth [7].

Although cgMLST is a widely used tool for investigating transmission and outbreaks of *P. aeruginosa*, there is no universally accepted cut-off for allelic differences that reliably defines outbreak-related isolates considering epidemiology. A cut-off of ≤ 11 distinct alleles is proposed for *P. aeruginosa* in general. However, in our last outbreak report, we discussed a clone-individual cut-off and suggested a cut-off of ≤ 3 allelic differences for *P. aeruginosa* ST111 which was also applicable to non-ST111 clusters [15]. Indeed, this cut-off was applicable to five highly related clusters (C1, C2, C4, C7, C8), all of which were epidemiologically linked to the same respective ward. The two CN-DTRPA clusters (C7 and C8) even showed overlapping ward stays, strengthening the evidence for possible interpatient transmission, though environmental sources should not be overlooked. Notably, the C1 (ST111) outbreak extended over a five-year period, with isolates differing by only ≤ 3 alleles, comparable to clusters C2 and C4 with a single allelic difference, but detected two years apart. This pattern suggests the presence of a low-mutator strain. In contrast, the C3 (ST273) outbreak evolved within a two-month period and remained within the ≤ 11-allele cut-off, consistent with a high-mutator phenotype. These findings argue for a more differentiated interpretation of cut-offs in outbreak definitions. However, prior research suggests that neither the genetic distance between clonal *P. aeruginosa* isolates nor the number of affected patients correlates proportionally with the spatiotemporal extent of an outbreak [24].

Hence, active screening for DTRPA is likely most effective when targeted at patients with risk factors for CP-DTRPA. Additionally, molecular detection tools with targeted PCR can be used for rapid CP-DTRPA detection, triggering subsequent IPC measures like environmental screening, ideally performed repeatedly and over extended periods to identify environmental reservoirs. Early identification of environmental reservoirs enables targeted interventions such as washbasin-free ICU designs, siphon replacement and regular siphon disinfection, which may further reduce environmental persistence [21]. In contrary, ABS strategies rather serve as supportive measures in managing CP-DTRPA infections with reported increased mortality [32].

Several limitations must be acknowledged. First, plasmids carrying resistance determinants may have been lost during laboratory process, potentially leading to underestimation of carbapenemase prevalence as seen in mixed CP-/CN-DTRPA C9 (ST357) cluster. Second, colony picking in routine diagnostics might not always capture the full diversity of resistant subpopulations. Third, changes in diagnostic workflows and interpretive criteria (e.g., EUCAST’s redefinition of ‘intermediate’ in 2019) may have influenced isolate selection and detection sensitivity. Fourthly, we performed short-read-WGS and hence, limiting differentiation between clonal spread from horizontal plasmid transfer of carbapenemases between unrelated STs. Fifthly, environmental sampling was not conducted on all affected wards, and only environmental CP-DTRPA isolates were sequenced, limiting conclusions regarding environmental acquisition of CN-DTRPA. Environmental sampling and sequencing for CN-DTRPA is hence recommended for future studies. Sixthly, as prior CSPA isolates were not sequenced, we cannot definitively confirm that all CN-DTRPA arose from pre-existing CSPA colonisation. Seventhly, the potential role of antimicrobial selection in the emergence of CN-DTRPA from CSPA-precolonisation remains hypothetical, as the available antibiotic exposure data were limited to cluster-associated patients and did not demonstrate a significant difference in meropenem exposure. Finally, our data is limited to a single centre whereas a multicentre study may deliver more data in similar settings.

## Conclusion

In summary, this study demonstrates that CN-DTRPA constitute the majority of DTRPA isolates and probably predominantly arise through antibiotic-driven resistance development in previously CSPA colonised patients explaining the ST diversity. In contrast, CP-DTRPA, although less frequent, were strongly associated with clusters and possible environmental transmission events. Our findings question the rationale of equal treatment of CP-DTRPA and CN-DTRPA in current international guidelines and rather support a differentiated IPC strategy in which isolation precautions and intensified screening are reserved for CP-DTRPA. Therefore, multicentre surveillance studies and surveillance of environmental CN-DTRPA should be performed to further investigate if isolation precautions and routine screening for CN-DTRPA may be unjustified and unlikely to provide additional benefit in preventing DTRPA transmissions.

## Supplementary Information

Below is the link to the electronic supplementary material.

Supplementary Material 1

Supplementary Material 2

## Data Availability

The assembled genomes of the 39 patients involved in clusters have been deposited to the public database (NCBI GeneBank database) under the BioProject no. PRJNA1453461.

## Abbreviations

cgMLST: Core genome multilocus sequence typing
CN-DTRPA: Carbapenemase-negative difficult-to-treat Pseudomonas aeruginosa
CP-DTRPA: Carbapenemase-producing difficult-to-treat Pseudomonas aeruginosa
CSPA: Carbapenem-susceptible Pseudomonas aeruginosa
DTRPA: Difficult-to-treat Pseudomonas aeruginosa
ICU: Intensive care unit
IPC: Infection prevention and control
P. aeruginosa: Pseudomonas aeruginosa
WGS: Whole genomse sequencing

## References

[1] Tamma PD, Heil EJ, Justo JA, Mathers AJ, et al. Infectious Diseases Society of America 2024 guidance in the treatment of antimicrobial-resistant gram-negative infections. Clin Infect Dis. 2024. 10.1093/cid/ciae403 39108079

[2] Livermore DM. Multiple mechanisms of antimicrobial resistance in *Pseudomonas aeruginosa*: our worst nightmare? Clin Infect Dis. 2002. 10.1086/338782 11823954

[3] del Barrio-Tofiño E, López-Causapé C, Oliver A. *Pseudomonas aeruginosa* epidemic high-risk clones and their association with horizontally acquired β-lactamases: 2020 update. Int J Antimicrob Agents. 2020. 10.1016/j.ijantimicag.2020.106196 33045347

[4] Büchler AC, Shahab SN, Severin JA, Vos MC. Voor In ‘t Holt AF. Outbreak investigations after identifying carbapenem-resistant *Pseudomonas aeruginosa*: a systematic review. Antimicrob Resist Infect Control. 2023. 10.1186/s13756-023-01223-1 PMC10068724 37013661

[5] Wendel AF, Malecki M, Mattner F, Xanthopoulou K, Wille J, Seifert H, et al. Genomic-based transmission analysis of carbapenem-resistant *Pseudomonas aeruginosa* at a tertiary care centre in Cologne (Germany) from 2015 to 2020. JAC Antimicrob Resist. 2022. 10.1093/jacamr/dlac057 PMC9122648 35611260

[6] Gill CM, Aktaş E, Alfouzan W, Bourassa L, Brink A, Burnham CD, Canton R, et al. The ERACE-PA Global Surveillance Program: ceftolozane/tazobactam and ceftazidime/avibactam in vitro activity against a global collection of carbapenem-resistant *Pseudomonas aeruginosa*. Eur J Clin Microbiol Infect Dis. 2021. 10.1007/s10096-021-04308-0 PMC8590662 34291323

[7] Pedersen T, Hesselberg Løvestad A, Ingebretsen A, Skutlaberg DH, Ås CG, Blomfeldt A. Molecular epidemiology of a nationwide *Pseudomonas aeruginosa* outbreak in Norway reveals multiple distinct clusters, including high-risk clone ST244. J Hosp Infect. 2025. 10.1016/j.jhin.2025.11.004 41271168

[8] World Health Organization. Guidelines for the prevention and control of carbapenem-resistant Enterobacteriaceae, *Acinetobacter baumannii* and *Pseudomonas aeruginosa* in health care facilities. Geneva: WHO; 2017. 29630191

[9] Sussenbach AE, Weterings V, Bathoorn E, Tielemans MJ, Ten Oever J, Visch B, et al. Dutch guideline for the prevention and control of multidrug-resistant organisms in the hospital setting: 2024 update. Antimicrob Resist Infect Control. 2025. 10.1186/s13756-025-01648-w PMC12595780 41204306

[10] Tacconelli E, Cataldo MA, Dancer SJ, De Angelis G, Falcone M, Frank U, et al. ESCMID guidelines for the management of infection control measures to reduce transmission of multidrug-resistant Gram-negative bacteria in hospitalized patients. Clin Microbiol Infect. 2014. 10.1111/1469-0691.12427 24329732

[11] KRINKO. Hygienemaßnahmen bei Infektionen oder Besiedlung mit multiresistenten gramnegativen Stäbchen. Bundesgesundheitsblatt Gesundheitsforschung Gesundheitsschutz. 2012. 10.1007/s00103-012-1549-5 23011096

[12] Wilson AP, Livermore DM, Otter JA, Warren RE, Jenks P, Enoch DA, et al. Prevention and control of multi-drug-resistant Gram-negative bacteria: recommendations from a joint working party. J Hosp Infect. 2016. 10.1016/j.jhin.2015.08.007 26598314

[13] Centers for Disease Contol and Prevention (CDC). MDRO Containment Strategy. 2024. Available from https://www.cdc.gov/healthcare-associated-infections/php/preventing-mdros/mdro-containment-strategy.html.

[14] Centers for Disease Control and Prevention (CDC). MDRO Prevention Strategies. 2024. Available from https://www.cdc.gov/healthcare-associated-infections/php/preventing-mdros/mdro-prevention-strategies.html.

[15] Jasuja JK, Klupp EM, Aepfelbacher M, Kampe KKW, Nentwich MF, Kluge S, Knobloch JK. Hidden transmissions of *Pseudomonas aeruginosa* ST111—the importance of continuous molecular surveillance. Antimicrob Resist Infect Control. 2025. 10.1186/s13756-025-01619-1 PMC12357456 40817071

[16] Carlsen E, Büttner H, Christner M, Franke G, Indenbirken D, Knobling B, et al. High burden and diversity of carbapenemase-producing *Enterobacterales* observed in wastewater of a tertiary care hospital in Germany. Int J Hyg Environ Health. 2022. 10.1016/j.ijheh.2022.113968 35390565

[17] Jolley KA, Bray JE, Maiden MCJ. A RESTful application programming interface for the PubMLST molecular typing and genome databases. Database (Oxford). 2017. 10.1093/database/bax060 PMC5550937 29220452

[18] Stanton RA, McAllister G, Daniels JB, Breaker E, Vlachos N, Gable P, et al. Development and application of a core genome multilocus sequence typing scheme for the health care-associated pathogen *Pseudomonas aeruginosa*. J Clin Microbiol 2020. 10.1128/JCM.00214-20 PMC7448633 32493782

[19] Rath A, Kieninger B, Fritsch J, Caplunik-Pratsch A, Blaas S, Ochmann M, et al. Whole-genome sequencing reveals two prolonged simultaneous outbreaks involving *Pseudomonas aeruginosa* high-risk strains ST111 and ST235 with resistance to quaternary ammonium compounds. J Hosp Infect. 2024;155–64. 10.1016/j.jhin.2024.01.009 38286239

[20] Feldgarden M, Brover V, Haft DH, Prasad AB, Slotta DJ, Tolstoy I, et al. Validating the AMRFinder tool and resistance gene database by using antimicrobial resistance genotype–phenotype correlations in a collection of isolates. Antimicrob Agents Chemother. 2019. 10.1128/AAC.00483-19 PMC6811410 31427293

[21] Catho G, Martischang R, Boroli F, Chraïti MN, Martin Y, Koyluk Tomsuk Z, et al. Outbreak of *Pseudomonas aeruginosa* producing VIM carbapenemase in an intensive care unit and its termination by implementation of waterless patient care. Crit Care. 2021. 10.1186/s13054-021-03726-y PMC8376114 34412676

[22] Pirzadian J, Persoon MC, Severin JA, Klaassen CHW, de Greeff SC, Mennen MG, et al. National surveillance pilot study unveils a multicenter, clonal outbreak of VIM-2-producing *Pseudomonas aeruginosa* ST111 in the Netherlands between 2015 and 2017. Sci Rep. 2021. 10.1038/s41598-021-00205-w PMC8545960 34697344

[23] Pham TM, Büchler AC, Voor in ’t holt AF, Severin JA, Bootsma MCJ, Gommers D, et al. Routes of transmission of VIM-positive *Pseudomonas aeruginosa* in the adult intensive care unit: analysis of 9 years of surveillance using a mathematical model. Antimicrob Resist Infect Control. 2022. 10.1186/s13756-022-01095-x PMC8981946 35379340

[24] Romano-Bertrand S, Virieux-Petit M, Mauffrey F, Senn L, Blanc DS. Defining a genomic threshold for investigating *Pseudomonas aeruginosa* hospital outbreaks. J Hosp Infect. 2025. 10.1016/j.jhin.2025.04.028 40355090

[25] Debast SB, van den Bos-Kromhout MI, de Vries-van Rossum SV, Abma-Blatter SEM, Notermans DW, Kluytmans JAJW, et al. Aquatic reservoir-associated outbreaks of multidrug-resistant bacteria: a hospital outbreak report of *Pseudomonas aeruginosa* in perspective from the Dutch national surveillance databases. J Hosp Infect. 2025. 10.1016/j.jhin.2025.05.024 40532976

[26] Simner PJ, Pitout JDD, Dingle TC. Laboratory detection of carbapenemases among Gram-negative organisms. Clin Microbiol Rev. 2024. 10.1128/cmr.00054-22 PMC11629623 39545731

[27] Moretti M, Vanstokstraeten R, Crombé F, Barbé K, Wybo I, Allard SD, et al. Five-year VIM-producing *Pseudomonas aeruginosa* outbreak in four Belgian ICUs, an investigation report (2019–2023). Am J Infect Control. 2024. 10.1016/j.ajic.2024.08.022 39218401

[28] Boyer A, Doussau A, Thiébault R, Venier AG, Tran V, Boulestreau H, et al. *Pseudomonas aeruginosa* acquisition in an intensive care unit: relationship between antibiotic selective pressure and patients’ environment. Crit Care. 2011. 10.1186/cc10026 PMC3221986 21306623

[29] Johnson JK, Smith G, Lee MS, Venezia RA, Stine OC, Nataro JP, et al. The role of patient-to-patient transmission in the acquisition of imipenem-resistant *Pseudomonas aeruginosa* colonization in the intensive care unit. J Infect Dis. 2009. 10.1086/605408 PMC3312466 19673646

[30] Raman G, Avendano E, Chan J, Merchant S, Puzniak L. Risk factors for hospitalized patients with resistant or multidrug-resistant *Pseudomonas aeruginosa* infections: a systematic review and meta-analysis. Antimicrob Resist Infect Control. 2018. 10.1186/s13756-018-0370-9 PMC6032536 29997889

[31] Boutin S, Scherrer M, Späth I, Kocer K, Heeg K, Nurjadi D. Cross-contamination of carbapenem-resistant Gram-negative bacteria between patients and the hospital environment in the first year of a newly built surgical ward. J Hosp Infect. 2024. 10.1016/j.jhin.2023.11.016 38081456

[32] Reyes J, Komarow L, Chen L, Ge L, Hanson BM, Cober E, et al. Global epidemiology and clinical outcomes of carbapenem-resistant *Pseudomonas aeruginosa* and associated carbapenemases (POP): a prospective cohort study. Lancet Microbe. 2023. 10.1016/S2666-5247(22)00329-9 PMC10016089 36774938

