# Supplementary material for: Retrospective analysis of the transmission of difficult-to-treat resistant Pseudomonas aeruginosa – genotype beats phenotype
Source: Antimicrob Resist Infect Control. 2026 Oct 30;15:134. doi: 10.1186/s13756-026-01851-3 (PMC13723103; doi:10.1186/s13756-026-01851-3)
Supplement: Supplementary file 1 [file 13756_2026_1851_MOESM1_ESM.docx]

**Supplementary Table 1** Overview of international guidelines available in English and/or German regarding infection prevention and control measure of multidrug-resistant organisms. The guidelines were analysed with regard to the differentiation between carbapenemase-producing (CP-) and carbapenemase-negative (CN-) difficult-to-treat resistant Pseudomonas aeruginosa (DTRPA). Where available, the evidence level was also listed. NA= not available

| **Origin/Country** | **Guideline** | **Year of publication** | **Differentiation between  CP-DTRPA and CN-DTRPA** | **Evidence level** |
| --- | --- | --- | --- | --- |
| Global | World Health Organization (WHO) | 2017 | No | NA |
| Dutch | Dutch guideline for the prevention and control of multidrug-resistant organisms in the hospital setting, 2024 update | 2024 | No | NA |
| Europe | ESCMID guidelines for the management of the infection control measures to reduce transmission of multidrug-resistant Gram-negative bacteria in hospitalized patients | 2014 | No | Moderate to low level evidence |
| Germany | Hygienemaßnahmen bei Infektionen oder Besiedlung mit multiresistenten gramnegativen Stäbchen Empfehlung der Kommission für Krankenhaushygiene und Infektionsprävention (KRINKO) beim Robert Koch-Institut (RKI) | 2012 | No | Recommendation based on limited evidence and expert consensus |
| UK | Prevention and control of multi-drug-resistant Gram-negative bacteria: recommendations from a Joint Working Party | 2016 | Yes | NA |
| USA | CDC – Healthcare-Associated Infection (HAIs) Prevention strategies | 2006 | Yes | Strongly recommended for implementation and strongly supported by well-designed experimental, clinical, or epidemiologic studies. |
